# Drug-Induced Liver Injury Following Co-ingestion of Veterinary Fenbendazole and Ivermectin for Prostate Cancer: A Case Report

**DOI:** 10.7759/cureus.108896

**Published:** 2026-05-15

**Authors:** Grace E Powderly, Kiley Hassevoort, Morgan Loy, Jamie Balonier, Corey Sievers

**Affiliations:** 1 Medical Education, A.T. Still University, Kirksville College of Osteopathic Medicine, Kirksville, USA; 2 Gastroenterology, Western Reserve Hospital, Cuyahoga Falls, USA

**Keywords:** alternative medical therapies, drug-induced liver injury (dili), fenbendazole, ivermectin treatment, prostate cancer treatment, self-administration

## Abstract

The self-administration of veterinary-grade anthelmintics, such as fenbendazole and ivermectin, has gained visibility in online communities due to unverified claims regarding potential anticancer effects. Despite these claims, these agents lack human regulatory approval due to unestablished safety profiles. We report a rare case of severe drug-induced liver injury (DILI) in a 65-year-old male with prostate cancer resulting from the concurrent ingestion of veterinary-grade fenbendazole and ivermectin. The patient presented to his primary care provider with a 2-week history of fatigue, jaundice, and abdominal pain following a 3-month regimen of alternating these agents daily at a dosage of “one squirt” (estimated to be 0.18 mg/kg of ivermectin and 0.98 mg/kg of fenbendazole) based on advice from online cancer support groups. Initial laboratory studies revealed profound transaminase elevations, with alanine aminotransferase (ALT) at 1764 U/L, aspartate aminotransferase (AST) at 1132 U/L, and a total bilirubin of 12.9 mg/dL. An R-value of 37.50 confirmed a hepatocellular injury pattern, while an extensive workup ruled out viral, autoimmune, and biliary etiologies. Following the complete cessation of both agents, transaminase levels decreased by 58% within 9 days and normalized within 6 weeks. A Roussel Uclaf Causality Assessment Method (RUCAM) score of 9 indicated a “highly probable” causal relationship between the anthelmintics and the acute liver injury. This case highlights the significant hepatotoxic risks associated with veterinary-grade therapies driven by medical misinformation. Overall, this case emphasizes the need for physicians to maintain a high index of suspicion for alternative therapies in oncology patients.

## Introduction

Fenbendazole and ivermectin are anthelmintic agents commonly used to treat animal parasitic infections. Researchers propose that these drugs possess antitumor effects against a variety of cancers. To induce antineoplastic effects, fenbendazole mimics the action of taxanes and vinca alkaloids by disrupting microtubule stability [1] while ivermectin targets multiple signaling pathways to inhibit cancer cell proliferation and induce apoptosis [2]. The use of fenbendazole against androgen-independent prostate cancer has been explored, with research showing decreased growth and proliferation of prostate cancer cells in in vivo models [3]. Although these anthelmintic drugs are considered promising anticancer agents, their use in humans lacks regulatory approval due to unknown safety profiles [1].

Ivermectin is currently approved for oral use in humans for treatment of onchocerciasis, strongylodiasis, and systemic filariasis, and topically for scabies [4]. In contrast, fenbendazole remains unapproved for human use in the United States and is restricted to the veterinary setting [5]. These agents possess distinct metabolic profiles. Ivermectin is primarily metabolized by CYP3A4 via hydroxylation and methylation, with secondary contributions from CYP3A5 and 2C9 [6]. In contrast, fenbendazole is metabolized primarily through hydrolysis by CYP2J2 and 2C19 [7]. Fenbendazole has also been shown to induce CYP1A enzymes. While the concurrent use of these agents is established as safe in veterinary practice under regulated dosing, there are no safety data to support the chronic, combined administration of these compounds in humans.

A recent case study demonstrated both an increase in liver transaminases and histological changes in the liver consistent with drug-induced liver injury (DILI) in correlation with the use and discontinuation of fenbendazole [8]. While ivermectin in approved doses is typically associated with a low risk of hepatotoxicity, it is classified by NCBI LiverTox with a Likelihood Score of D, indicating it as a rare but possible cause of clinically apparent liver injury [9]. Case reports have specifically described a hepatocellular pattern of injury following ivermectin use [10-12]. Patients who self-prescribe veterinary formulations may exceed the established safety profile by transitioning from standard single-dose regimens to chronic daily administration. Given that both medications have shown independent evidence of potential liver injury, their unregulated concurrent use may place patients at risk for a cumulative hepatotoxic insult.

While national epidemiological data on anthelmintic misuse for cancer are emerging, institutional reports indicate a persistent trend. A 2026 single-institutional experience from MD Anderson Cancer Center noted that self-reported use of ivermectin and fenbendazole has "organically" persisted since the COVID-19 pandemic, particularly among patients with prostate, colorectal, and metastatic diseases [13]. This trend may be fueled by anecdotal reports in online communities, such as those reviewed by Nguyen et al., where perceived tumor reduction was described in patients self-administering fenbendazole alongside conventional therapies [1]. However, the use of these veterinary-grade formulations presents a measurable clinical challenge, as several studies now document serious adverse effects, including hepatotoxicity in human patients [8,10,14-16].

In today’s digital age, patients often seek medical information online and on social media. The presence of health misinformation online can impact medical decision-making and the clinician-patient relationship [17,18]. Recent narrative reviews have highlighted that online misinformation frequently promotes unverified claims regarding the safety and efficacy of alternative treatments, often leveraging emotional narratives that bypass traditional clinical evidence [19]. A recent case in pediatric osteosarcoma treatment highlights the impact of misinformation surrounding ivermectin and the off-label uses of non-proven medications for cancer treatment [20]. In regards to ivermectin, serious adverse effects, involving neurological and hepatic effects like encephalopathy, have been reported in patients who ingest this drug in the absence of a systemic parasitic infection [21]. While there is a general lack of epidemiological evidence on this issue, the presence of a multitude of cases, with a variety of patient populations and suspected indications of use, warrants further investigation into this current phenomenon. This article reports the case of a patient with prostate cancer with “highly probable” DILI after the self-administration of veterinary fenbendazole and ivermectin, agents claimed to possess anti-cancer properties on various online platforms.

## Case presentation

**Figure 1:**
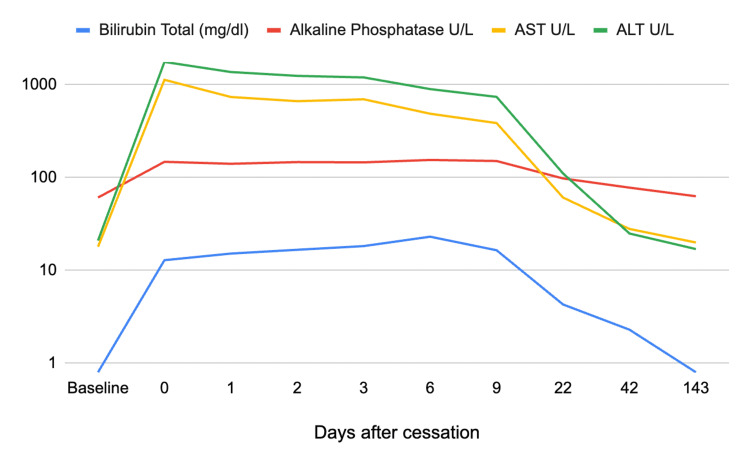
Graphical representation of longitudinal trends in liver function tests after cessation of fenbendazole and ivermectin AST: Aspartate Aminotransferase; ALT: Alanine Aminotransferase

A 65-year-old male with a history of prostate cancer presented to his primary care provider with worsening fatigue, vague abdominal pain, and yellowing of the skin. He had fatigue and exercise intolerance for two weeks, with the development of abdominal pain, nausea, and vomiting in the past five days. He produced pale stools and dark urine for three days and noticed yellowing of his skin around this time. He denied pruritus. The patient reported no history of liver disease and denied any recent travel, illnesses, tobacco use, or weight loss. Alcohol use included three to five beers each week. His only reported medications were veterinary-grade fenbendazole and ivermectin. Upon further questioning, the patient revealed he was undergoing active surveillance for his prostate cancer, supplemented with three months of oral ingestion of veterinary-grade fenbendazole and ivermectin for perceived anticancer effects as suggested by online prostate cancer support groups. The patient self-administered the veterinary-grade anthelmintics on an alternating daily schedule. Quantitative exposure was estimated based on a reported volume of approximately 1 mL per administration. Using standard veterinary concentrations (1.87% ivermectin and 10% fenbendazole) and the patient’s weight of 102.27 kg, the estimated doses were 0.18 mg/kg of ivermectin and 0.98 mg/kg of fenbendazole. Symptom onset was approximately 90 days after the introduction of these two agents. Physical exam showed pertinent findings of scleral icterus, jaundice, and mild abdominal distention without abdominal tenderness. Initial outpatient laboratory results revealed elevated total bilirubin 12.9 mg/dL, alkaline phosphatase (ALP) 148 U/L, aspartate aminotransferase (AST) 1132 U/L, and alanine aminotransferase (ALT) 1764 U/L, values that were previously normal at prior visits (Table 1) (Figure 1). Laboratory evaluation was also notable for a normal serum ammonia level of 36 umol/L, correlating with the absence of hepatic encephalopathy upon presentation. The patient’s international normalized ratio (INR) was elevated at 1.7 (reference 0.8-1.1), and prothrombin time (PT) was elevated at 20.0 (reference 9.4-12.5). At presentation, the calculated R-value was 37.50, identifying a profound hepatocellular pattern of liver injury. These laboratory values resulted the following day, and he was sent to the emergency department for further workup with subsequent admission for hyperbilirubinemia.

**Table 1:** Longitudinal trends in liver function tests after cessation of fenbendazole and ivermectin ALP: Alkaline Phosphatase; AST: Aspartate Aminotransferase; ALT: Alanine Aminotransferase

Post-Cessation Day	Bilirubin, Total (mg/dL)	ALP (U/L)	AST (U/L)	ALT (U/L)
Baseline	0.8	61	18	21
Day 0	12.9	148	1132	1764
Day 1	15.2	141	739	1373
Day 2	16.7	147	666	1248
Day 3	18.3	146	698	1201
Day 6	23.1	155	488	899
Day 9	16.5	151	387	741
Day 22	4.3	98	61	111
Day 42	2.3	78	28	25
Day 143	0.8	63	20	17
Normal Range	≤1.2	35-129	≤40	≤41

Upon admission, the gastrointestinal service was consulted for the workup of acute liver injury. The initial differential diagnosis included viral hepatitis, neoplasm, autoimmune hepatitis, alcoholic hepatitis, Wilson's disease, hemochromatosis, Budd-Chiari syndrome, biliary obstruction, drug-induced liver injury, etc. Serum alcohol level on admission was undetectable (≤ 9.9 mg/dL). Additionally, a serum acetaminophen level was obtained and was undetectable (<5 µg/mL), ruling out acute acetaminophen-induced hepatotoxicity. Viral hepatitis panel, ceruloplasmin, anti-smooth muscle antibodies, and Epstein-Barr Virus Viral Capsid Antigen Immunoglobulin M (EBV VCA IgM) were all unremarkable.

To evaluate for biliary and vascular etiologies of jaundice, a comprehensive imaging workup was performed. A right upper quadrant ultrasound and MRCP demonstrated a normal-sized liver (14 cm) and a patent biliary tree without evidence of choledocholithiasis or ductal dilatation. Computed tomography of the abdomen and pelvis with contrast and ultrasound both noted a stable 3 cm left hepatic lobe cyst and nonspecific gallbladder wall thickening (8 mm). In the absence of cholelithiasis or hyperemia, this thickening was interpreted as reactive to the acute hepatic inflammation. Vascular patency was confirmed with hepatopetal portal vein flow, effectively ruling out Budd-Chiari syndrome and portal vein thrombosis. 

The initial management of this patient included counseling regarding the complete cessation of fenbendazole and ivermectin use. The patient was also advised to avoid hepatotoxic drugs such as acetaminophen. No pharmacological treatments were initiated. Inpatient observation was continued for three days to monitor liver function, which showed decreasing values of ALT and AST throughout the hospital stay. The patient remained hemodynamically stable and was consequently discharged with the presumed diagnosis of liver injury secondary to hepatotoxic drugs, with planned outpatient liver function tests. Laboratory values showed a 58% decline in ALT levels after nine days of complete cessation of these drugs (Table 1) (Figure 1). Liver transaminases normalized within six weeks. Complete resolution of his liver injury was documented on his most recent laboratory evaluation (Table 1). Ultimately, the presumed diagnosis of drug-induced liver injury was supported by rapid improvement after discontinuation of the offending agents, fenbendazole and ivermectin. To assess causality, the updated Roussel Uclaf Causality Assessment Method (RUCAM) score was calculated [22]. The patient’s score of 9 indicates a highly probable (RUCAM ≥ 9) relationship between the anthelmintic co-ingestion and the subsequent liver injury (Table 2) [22].

**Table 2:** Roussel Uclaf Causality Assessment Method (RUCAM) for suspected drug-induced liver injury Source: [22] ALT: Alanine Aminotransferase

RUCAM Causality Assessment		
Time to Onset	+2	Symptoms/jaundice began ~90 days after starting the regimen (Range: 5-90 days).
Course (Improvement after Cessation)	+3	ALT decreased by 58% (>50%) within 9 days of stopping the drugs
Risk Factors	+1	Patient age ≥ 55 years (+1); Alcohol use was ﹤2 drinks/day (0)
Concomitant Drugs	+0	No other new medications or known hepatotoxins were started in the previous 90 days
Exclusion of Other Causes	+2	Extensive workup (viral, autoimmune, biliary, metabolic) was negative.
Previous Information on Drugs	+1	Known hepatotoxicity in the literature
Total	9	Interpretation: Highly Probable

## Discussion

DILI is a leading cause of acute liver failure in the United States, requiring rapid identification and cessation of the offending agent to avoid disease progression and liver transplantation [23]. Most cases of DILI include symptoms similar to other forms of hepatitis such as fatigue, nausea, jaundice, malaise, or pruritus. To make the diagnosis of DILI, infectious, autoimmune, and other forms of liver disease must be ruled out. Due to differing drug mechanisms, symptom onset may occur at different times depending on the specific drug ingested [23]. Improvement of symptoms after drug cessation may help confirm the diagnosis; similarly, symptom onset after re-challenge may be confirmatory. The main challenge of a DILI diagnosis involves establishing a causal relationship; clinical scales, such as the updated RUCAM, have been created to help establish a diagnosis of DILI [22]. 

In this case, the patient’s clinical symptoms of jaundice and elevated transaminases were suggestive of a hepatocellular pattern of injury. To assess this, the R-value was calculated by taking the ratio of ALT to ALP relative to their respective upper limits of normal. According to standardized DILI classification criteria, an R-value ≥ 5.0 confirms a hepatocellular pattern of injury [22]. This patient’s R-value was 37.50, more than 7 times the diagnostic threshold; this directed the diagnostic workup towards exclusion of viral, metabolic, and autoimmune causes. This patient met Hy’s Law criteria with AST and ALT elevations > 3x the upper limit of normal, without significant elevations in ALP (> 2x upper limit of normal). Satisfying these criteria increases a patient’s risk of mortality due to DILI by more than 10% [24]. While the patient reported a baseline alcohol intake of three to five beers weekly, his serum alcohol level on admission was undetectable, and his biochemical profile was highly uncharacteristic of alcohol-induced liver disease. Whereas alcoholic hepatitis typically presents with an AST/ALT ratio > 2:1 and transaminases rarely exceeding 500 U/L, this patient exhibited a hepatocellular pattern with ALT (1764) significantly higher than AST (1132 U/L). In addition to these criteria, other etiologies of liver injury were explored and ruled out clinically during the diagnostic process. Furthermore, the patient’s serum acetaminophen level was undetectable upon admission, ruling out the most common pharmacologic cause of acute liver injury in the United States. Although a broad urine toxicology screen was not performed, the clinical combination of a negative acetaminophen level, a negative history of other supplement use, and the rapid recovery upon drug cessation strongly excludes other toxic etiologies.

To further establish causality, the updated RUCAM score was calculated, which is the current gold standard assessment for a DILI diagnosis [22]. Due to the relationship between drug ingestion and symptom onset, the exclusion of differential etiologies, and the rapid normalization after drug discontinuation, the patient’s updated RUCAM score was calculated at 9. Transparency in scoring is provided in Table 2. The patient’s latency period (the time from initiation of the anthelmintic regimen to the onset of jaundice) was approximately 90 days. This timeline is highly characteristic of idiosyncratic DILI (+2 points), where hepatic injury often occurs within the first one to three months after onset of exposure [25]. Furthermore, after drug cessation, the 58% decline in ALT levels within 9 days of drug cessation meets the RUCAM criteria for a highly probable positive dechallenge (50% drop within 8 days) (+3 points) [22]. Additionally, the exclusion of all alternative etiologies added +2 points, while the patient’s age contributed +1 point. Hepatotoxicity has been documented in peer-reviewed literature for both ivermectin [9,10] and fenbendazole [16,26], adding +1 point to the RUCAM score. The total RUCAM score of 9 establishes a “highly probable” causal relationship between the ingestion of veterinary-grade fenbendazole/ivermectin and the acute liver injury rooted in objective, validated criteria rather than clinical suspicion alone. RUCAM scores are interpreted as follows: ≥ 9, highly probable; 6-8, probably; 3-5, possible; 1-2, unlikely; and ≤ 0, excluded [22].

A biopsy was avoided because the diagnosis was clinically evident after discontinuation of the fenbendazole and ivermectin, sparing the patient an unnecessary, invasive procedure. While a formal drug re-challenge could further strengthen causality between anthelmintic ingestion and liver injury, it was deemed ethically contraindicated given the severity of the patient’s initial hepatocellular injury (ALT 1764 U/L). In this case, the rapid improvement of transaminases within six weeks of cessation supports the "highly probable" RUCAM score in the absence of re-challenge data.

This "highly probable" clinical assessment is further supported by a quantitative analysis of the patient’s drug exposure. Based on the reported administration of approximately 1 mL "squirts" of veterinary-grade paste, the estimated weight-based dosages were 0.18 mg/kg of ivermectin and 0.98 mg/kg of fenbendazole on alternating days. While the ivermectin dose falls within the FDA-approved standard human therapeutic range (0.15-0.2 mg/kg) [4], there is currently no FDA-approved human dose for fenbendazole [5]. Fenbendazole is strictly approved as a veterinary anthelmintic, with no current FDA or EMA approval for human use for any indication [5]. Toxicological data suggest that veterinary doses typically exceed the estimated human acceptable daily intake by more than 100-fold [27]. In this case, the patient’s self-administration of concentrated veterinary paste likely surpasses the established safety margins, suggesting a pharmacological basis for the observed liver injury. This increased exposure, combined with the "highly probable" RUCAM score of 9, suggests that the cumulative frequent administration of these agents may have triggered the acute hepatic insult.

The nature of this liver injury requires a distinction between intrinsic and idiosyncratic hepatotoxicity. The use of concentrated veterinary pastes exceeding the human safety margins suggests a component of intrinsic toxicity; however, the 90-day latency and profound transaminase elevation are more characteristic of idiosyncratic DILI, which is often mediated by a patient’s unique genetic profile. Specifically, certain human leukocyte antigen (HLA) alleles have been strongly connected to the risk of liver injury from various drugs, as they may facilitate an inappropriate T-cell-mediated immune response against the drug [25]. Although HLA typing was not performed in this case, the patient may have exhibited a genetic predisposition or metabolic polymorphism (in the CYP3A4 or CYP2C19 pathways) that lowered his threshold for hepatic injury. While the concentrated doses may have exerted an intrinsic metabolic stress, the severity of transaminases suggests a dual-mechanism injury with idiosyncratic vulnerability.

The use of fenbendazole has been linked to hepatotoxicity in humans at doses as low as 1 gram per day [16]. The hepatotoxic potential of these anthelmintic drugs aligns with a sparse body of literature regarding unregulated use in humans. Although Nguyen et al. report cases of tumor reduction after fenbendazole administration [1], this case highlights the significant adverse effects that may accompany unregulated use. This patient’s presentation mirrors a case report by Sidhu et al., which described a case of ivermectin-induced liver injury after self-injection with horse-strength ivermectin for COVID-19 prophylaxis [10]. Similarly, Thakurdesai et al. have documented a case of severe DILI associated with unregulated use of veterinary-grade fenbendazole for perceived anti-cancer effects [16]. This case is particularly noteworthy when compared to a similar case described by Chaturvedi et al., which reported fenbendazole-induced DILI in a metastatic breast cancer patient [8]. Compared to these cases with an isolated drug of causation, our patient’s injury (ALT 1764 U/L) was markedly more severe. This presentation may reflect a cumulative hepatotoxic insult resulting from the concurrent ingestion of both ivermectin and fenbendazole. Given that both agents possess independent evidence of potential liver injury, their unregulated dual administration may impose a heightened metabolic stress on the liver, exceeding the threshold of injury seen with single-agent exposure.

Furthermore, this case illustrates the potential impacts associated with the accessibility of unverified medical claims online, as described by Roozenbeek et al. and Patrick et al. [17,18]. This patient’s clinical decisions were influenced by anecdotal reports in online support groups encouraging the use of these anthelmintic drugs as alternative cancer therapies. This becomes particularly hazardous when involving veterinary-grade formulations, which may possess concentrations unsafe for human consumption. Ultimately, this reinforces the necessity of patient-centered communication and thorough history-taking. Providers should be aware that patients may seek alternative treatment options, and they may be hesitant to disclose the use of these treatments. Creating an honest, open, and nonjudgmental environment is essential to uncover the use of non-prescribed medications and supplements.

## Conclusions

This case illustrates the profound hepatotoxic risk associated with the off-label, concurrent self-administration of veterinary-grade fenbendazole and ivermectin. This “highly probable” RUCAM score of 9 and the rapid clinical recovery upon drug cessation emphasize the necessity of considering unregulated anthelmintics in the differential diagnosis of idiopathic liver injury. To improve early detection, clinicians should integrate specific screening questions regarding off-label medication use into routine histories. Establishing a non-judgmental, patient-centered environment is vital to encouraging the disclosure of these therapies, allowing providers to educate patients about the lack of established human safety and the potential for severe adverse effects.

The increasing prevalence of such cases, often driven by unverified online claims, warrants a shift in public health policy. Legislative measures to restrict over-the-counter access to veterinary medications not FDA-approved for human use, such as fenbendazole, should be explored to prevent continued misuse. Although research into the antineoplastic potential of these agents is ongoing, current clinical evidence suggests that the risk of unregulated use outweighs the perceived benefits. Given the broad dispersion of these cases, a multi-institutional retrospective study is needed to quantify the epidemiological burden of this phenomenon and identify the sources of medical misinformation influencing patient decisions.
